# Preoperative malnutrition is a risk factor for intraoperative hypotension in high-risk surgical patients: a propensity score–matched cohort study

**DOI:** 10.1186/s13741-025-00620-x

**Published:** 2025-11-19

**Authors:** Zhengzhen Huang, Chen Wang, Meimei Zhu, Ziyu Zhu, Xiaoyong Miao, Ying Yao, Jianping Cao, Yan Li

**Affiliations:** Department of Anesthesiology, Navy Medical Center, Naval Medical University, Shanghai, China

**Keywords:** Malnutrition, Intraoperative hypotension, High-risk surgical procedures, Perioperative care

## Abstract

**Background:**

Intraoperative hypotension (IOH) is associated with adverse outcomes in high-risk surgical patients. Preoperative malnutrition may increase susceptibility to IOH, but evidence regarding its association with IOH assessed through multiple dimensions remains limited. This study aimed to evaluate the association between malnutrition and IOH.

**Methods:**

This retrospective cohort study included 1504 adult patients who underwent elective high-risk non-cardiac surgery under general anesthesia. Nutritional status was assessed using the Global Leadership Initiative on Malnutrition (GLIM) criteria. IOH was evaluated across four dimensions: incidence, cumulative duration, proportion of anesthesia time in IOH, and lowest mean arterial pressure (MAP), based on both absolute (MAP < 65 mmHg) and relative (≥ 20% reduction from baseline) thresholds. The proportion of time in relative IOH, which demonstrated the most clinically meaningful difference, was pre-specified as the primary outcome for multivariable analysis. Associations were examined using propensity score matching and multivariable beta regression analysis.

**Results:**

Compared to non-malnourished patients, malnourished individuals had a significantly greater proportion of time in relative IOH (0.48 ± 0.16 vs. 0.39 ± 0.17, Cohen’s *d* = 0.54, *P* < 0.001), along with longer IOH duration and lower nadir MAP. Absolute IOH metrics showed no significant group differences. Malnutrition remained independently associated with increased relative IOH after adjustment for relevant covariates (*P* < 0.001). Subgroup and sensitivity analyses confirmed the robustness of these findings.

**Conclusions:**

Preoperative malnutrition is independently associated with increased intraoperative hemodynamic instability when assessed by relative blood pressure thresholds. These findings underscore the importance of incorporating nutritional risk into perioperative risk stratification and highlight the need for prospective studies to validate these associations.

**Supplementary Information:**

The online version contains supplementary material available at 10.1186/s13741-025-00620-x.

## Background

Malnutrition is a prevalentyet frequently underrecognized condition among hospitalized patients. It is associated with an increased risk of infection, prolonged hospitalization, and delayed recovery (Bañuls et al. 2019; Brock et al. 2016; Cai et al. 2023). Beyond its established effects on immune function and wound healing, malnutrition may also lead to hypoalbuminemia and endothelial dysfunction (Cederholm et al. 2024; Czapla et al. 2022). These alterations might impair perioperative hemodynamic stability by reducing circulating blood volume, disrupting the regulation of vascular tone, and compromising vascular structural integrity (de Luis et al. 2014; Glassman et al. 2025).

Intraoperative hypotension (IOH), a common manifestationof such instability, has been consistently associated with adverse postoperative outcomes, including cognitive dysfunction, myocardial injury, and acute kidney injury (Gregory et al. 2021; Jensen et al. 2025; Jung et al. 2024; Kalezic et al. 2013; Kocyigit et al. 2021). Recent evidence focusing on postoperative delirium further suggests that the duration and severity of IOH—rather than its mere occurrence—may have a more profound impact on the development of delirium, especially among patients undergoing prolonged or high-risk surgical procedures (Lai et al. 2022). This also suggests that the assessment of IOH may benefit from incorporating its duration and severity, in addition to its occurrence.

Although several physiological mechanisms—such as endothelial dysfunction—may link malnutrition to impaired hemodynamic stability, and clinical evidence from specific populations (including cardiac surgery patients, older adults with orthostatic intolerance, and obstetric patients) suggests that nutritional status can significantly affect circulatory regulation (Lin et al. 2021; Lubrano et al. 2024; Maiwall et al. 2022), its contribution in the broader surgical population undergoing general anesthesia remains insufficiently defined. Further investigation is warranted to determine whether preoperative nutritional status is associated with IOH in this context. Establishing this relationship may also enable earlier identification of at-risk patients and inform the development of targeted perioperative management strategies.

This study aimed to examine the association between preoperative malnutrition and IOH in patients undergoing elective high-risk surgery under general anesthesia. In order to achieve this, IOH was assessed across multiple clinically relevant dimensions using both absolute and relative thresholds, and propensity score–matched analysis was performed to minimize baseline confounding.

## Methods

### Study design and setting

This retrospective cohort study was conducted at a tertiary medical center, reviewing patients who underwent elective high-risk surgery under general anesthesia between December 2022 and December 2024.

### Ethical approval

Ethical approval (Approval No. AF-HEC-001) was granted by the institutional ethics committee on January 6, 2025. Written informed consent was waived due to the retrospective nature of the study and the anonymized nature of routinely collected clinical data. No identifiable patient information or images were used. All procedures were conducted in compliance with institutional ethical standards and the Declaration of Helsinki (2013 revision).

### Study cohort

Patients aged 18 years or older undergoing elective high-risk surgical procedures classified as Major or Complex Major (Johns Hopkins surgical risk classification) under general anesthesia at our institution from December 2022 to December 2024 were retrospectively identified from the institutional clinical database. Exclusion criteria included (1) end-stage renal disease (estimated glomerular filtration rate [eGFR] < 15 mL/min or long-term dialysis dependence); (2) severe cardiac dysfunction (New York Heart Association [NYHA] Class III–IV); (3) clinically significant autonomic dysfunction (detailed criteria provided in Supplementary Material 1); and (4) absence of intraoperative invasive arterial blood pressure (IABP) monitoring.

### Data collection and definitions

Clinical data were retrospectively extracted from electronic medical records by the study investigators using a standardized protocol. Age, sex, body mass index (BMI), American Society of Anesthesiologists (ASA) physical status classification, and comorbidities (hypertension, diabetes mellitus [DM], and chronic kidney disease [CKD]) were documented as baseline characteristics. Preoperative mean arterial pressure (MAP) was recorded as the baseline value.

Intraoperative variables included surgical type, laparoscopic surgery (yes/no), surgical position, surgery duration, total fluid intake, fluid management grade (low: < 5 mL/kg/h; moderate: ≥ 5 and < 10 mL/kg/h; high: ≥ 10 mL/kg/h), and estimated blood loss.

Preoperative nutritional and inflammatory status was assessed using laboratory markers, including serum albumin, CRP, lymphocyte count, and hemoglobin levels. These parameters were obtained through standard preoperative testing and documented in the patients’ medical records.

To fit the retrospective nature of this study, we applied the latest GLIM criteria adapted specifically for Asian populations, based on a Delphi consensus approach (Modir eta al., 2021; Olson et al., 2023). Malnutrition was diagnosed if patients met at least one phenotypic and one etiologic criterion:

- Phenotypic criteria: Low BMI (BMI < 18.5 kg/m^2^ for patients < 70 years; BMI < 20 kg/m^2^ for patients ≥ 70 years) or serum albumin < 35 g/L.
- Etiologic criteria: Evidence of systemic inflammation, defined as either (1) the presence of a chronic inflammatory condition (e.g., malignancy, chronic kidney disease, rheumatoid arthritis) or (2) a C-reactive protein (CRP) level > 5 mg/L. CRP elevation was interpreted as reflecting low-grade chronic inflammation in the absence of acute infection or sepsis, which were excluded.

Patients meeting at least one criterion from each domain were classified as malnourished (Malnutrition: Yes); all others were considered nutritionally normal (Malnutrition: No).

All required variables (exposures, covariates, and outcomes) were available for every patient as part of mandatory institutional assessments; thus, no missing data were present in the analytic dataset, and imputation was not required.

### Study outcomes

The primary outcome was IOH, assessed using both absolute and relative thresholds based on IABP monitoring. Although IABP provides continuous real-time measurements, for standardization and consistency, MAP values recorded at 5-min intervals in the anesthesia information system were used for analysis.

- Absolute IOH: Defined as an intraoperative MAP < 65 mmHg any recorded time point during surgery (Panwar et al. 2020).
- Relative IOH: For normotensive patients, defined as a ≥ 20% reduction from baseline MAP; for hypertensive patients, defined as a ≥ 20% reduction from baseline and an intraoperative MAP < 90 mmHg (Raynor et al. 2023). All hypotension criteria were applied to MAP values recorded at discrete 5-min intervals from the invasive arterial pressure monitor.

IOH severity and burden were quantified using the following parameters:

1. Incidence—whether the patient experienced at least one hypotensive event;
2. Duration—the total number of minutes with MAP below the IOH threshold;
3. Proportion—the percentage of total anesthesia time spent in hypotension, defined as the number of time points (recorded every 5 min) in which the MAP met the IOH criteria, divided by the total number of recorded time points during anesthesia;
4. Lowest MAP—the minimum recorded MAP during the procedure.

For analysis, each qualifying data point was assumed to represent a 5-min hypotensive period, consistent with the data recording frequency.

### Statistical methods

Frequencies (*n*) and percentages (%) were used to summarize categorical variables, and comparisons were performed using the chi-square test with Cohen’s *h* as the effect size (ES). For normally or approximately normally distributed continuous variables, data were presented as mean ± standard deviation (Mean ± SD) and assessed using Student’s *t*-test, with Cohen’s *d* as the effect size. Non-normally distributed data (absolute skewness > 1 or absolute kurtosis > 2) were expressed as median (interquartile range, IQR) and compared using the Mann–Whitney *U* test, with Cliff’s delta as the effect size.

To reduce selection bias and improve statistical efficiency, we employed propensity score matching (PSM). Specifically, nearest-neighbor matching without replacement was conducted using the logit-transformed propensity score, with a caliper width of 0.2 standard deviations. Preoperative covariates included in the propensity model were selected a priori based on their clinical relevance to both nutritional status and intraoperative hemodynamic vulnerability. To avoid inappropriate adjustment for post-exposure or intermediate variables, only baseline characteristics available prior to surgery were used in the matching process. This approach ensured temporal alignment between exposure and covariates, thereby reducing the risk of collider bias and reverse causation. Importantly, variables directly related to the diagnosis of malnutrition—such as BMI, serum albumin, and CRP—were intentionally excluded from the propensity score model to avoid over-adjustment or diagnostic circularity. The optimal matching ratio for primary analysis was determined based on overall balance and sample efficiency, as detailed in the “Results” section.

Among the IOH dimensions, the proportion of anesthesia time spent in relative IOH was a priori designated as the primary outcome for exploratory multivariable analyses. This variable was chosen a priori because its continuous scale provides higher resolution and sensitivity in quantifying cumulative hemodynamic burden, and it has strong clinical interpretability.

For exploratory multivariable analysis, least absolute shrinkage and selection operator (LASSO) regression (L1 regularization within a generalized linear model framework, using cross-validation for optimal parameter selection) was employed for variable selection. A beta regression model was then constructed to analyze the proportional outcome.

Model performance was evaluated using multiple criteria: model fit was assessed via the Akaike Information Criterion (AIC) and Bayesian Information Criterion (BIC), explanatory power was measured by Pseudo *R*^2^, and predictive accuracy was assessed using the Brier Score (optimal value < 0.1). To ensure robustness, stratified analyses were conducted based on surgical type and hypertension status, and a sensitivity analysis was performed using an inverse probability of treatment weighting (IPTW) dataset to validate the model’s applicability.

All statistical tests were two-tailed, with *P* < 0.05 considered statistically significant. Data analysis was performed in R (version 4.4.1).

## Results

### Study participants and matching strategy

A total of 1504 patients met the inclusion criteria, comprising 246 with malnutrition and 1258 without (Supplementary Material 2). The cohort had a mean age of 64.8 years, with 50.4% male patients, and most procedures involved thoracic, orthopedic, abdominal, or urologic surgery. Baseline demographic and perioperative characteristics of the overall cohort are summarized in Table 1.

**Table 1:** Baseline demographic and perioperative characteristics of patients and group balance before and after PSM

Variables	Before matching			After matching		
	Malnutrition	Malnutrition	SMD	Malnutrition	Malnutrition	SMD
	Yes (*n* = 246)	No (*n* = 1258)		Yes (*n* = 246)	No (*n* = 738)	
Age	64.83 ± 12.38	61.85 ± 12.26	0.243	64.83 ± 12.38	63.83 ± 12.09	0.081
Sex			−0.054			0.003
Female	122 (49.6)	658 (52.3)		122 (49.6)	367 (49.7)	
Male	124 (50.4)	600 (47.7)		124 (50.4)	371 (50.3)	
BMI (kg/m^2^)	20.95 ± 3.31	24.31 ± 2.82	−1.156	20.95 ± 3.31	24.19 ± 2.81	−1.102
ASA			0.456			0.141
II	147 (59.8)	1008 (80.1)		147 (59.8)	491 (66.5)	
III	99 (40.2)	250 (19.9)		99 (40.2)	247 (33.5)	
Hypertension			0.136			0.017
No	153 (62.2)	864 (68.7)		153 (62.2)	465 (63.0)	
Yes	93 (37.8)	394 (31.1)		93 (37.8)	273 (37.0)	
DM			0.061			0.032
No	201 (81.7)	1057 (84.0)		201 (81.7)	612 (82.9)	
Yes	45 (18.3)	201 (16.0)		45 (18.3)	126 (17.1)	
CKD			0.029			0.025
No	239 (97.2)	1228 (97.6)		239 (97.2)	720 (97.6)	
Yes	7 (2.8)	30 (2.4)		7 (2.8)	18 (2.4)	
Baseline MAP (mmHg)	107.46 ± 11.28	105.54 ± 10.96	0.173	107.46 ± 11.28	106.69 ± 11.17	0.068
Albumin (g/L)	33.40 ± 3.09	42.48 ± 4.43	−2.141	33.40 ± 3.09	42.63 ± 4.42	−2.235
CRP (mg/L)	6.28 (3.62,7.80)	4.19 (2.45,6.14)	0.781	6.28 (3.62,7.80)	4.28 (2.47,6.20)	0.884
Lymphocyte count (× 10^9^/L)	1.50 ± 0.49	1.55 ± 0.51	−0.099	1.50 ± 0.49	1.56 ± 0.51	−0.116
Hemoglobin (g/L)	126.44 ± 16.65	126.47 ± 17.81	−0.002	126.44 ± 16.65	126.62 ± 17.70	−0.010
Surgery type			0.097			0.011
Abdominal	84 (34.1)	396 (31.5)		84 (34.1)	245 (33.2)	
Orthopedic	61 (24.8)	358 (28.5)		61 (24.8)	189 (25.6)	
Thoracic	82 (33.3)	395 (31.4)		82 (33.3)	246 (33.3)	
Urological	19 (7.7)	109 (8.7)		19 (7.7)	58 (7.9)	
Laparoscopic surgery			0.011			0.016
No	64 (26.0)	321 (25.5)		64 (26.0)	187 (25.3)	
Yes	182 (74.0)	937 (74.5)		182 (74.0)	551 (74.7)	
Surgical position			0.091			0.024
Supine	18 (7.3)	100 (7.9)		18 (7.3)	56 (7.6)	
Trendelenburg	43 (17.5)	241 (19.2)		43 (17.5)	138 (18.7)	
Reverse Trendelenburg	50 (20.3)	240 (19.1)		50 (20.3)	140 (19.0)	
Lithotomy	33 (13.4)	159 (12.6)		33 (13.4)	100 (13.6)	
Lateral decubitus	97 (39.4)	479 (38.1)		97 (39.4)	284 (38.5)	
Prone	5 (2.0)	39 (3.1)		5 (2.0)	20 (2.7)	
Surgery duration (mins)	180.14 ± 33.13	174.41 ± 32.09	0.176	180.14 ± 33.13	178.46 ± 31.26	0.053
Anesthesia duration (mins)	194.70 ± 33.09	189.52 ± 32.75	0.157	194.70 ± 33.09	193.50 ± 31.60	0.037
Sufentanil dosage (μg)	45.04 ± 6.44	49.69 ± 6.69	−0.707	45.04 ± 6.44	49.38 ± 6.69	−0.656
Remifentanil dosage (mg)	1.45 ± 0.25	1.42 ± 0.25	0.132	1.45 ± 0.25	1.45 ± 0.24	0.010
Propofol dosage (mg)	389.02 ± 66.22	379.03 ± 65.77	0.151	389.02 ± 66.22	387.02 ± 63.42	0.031
Total fluid intake (mL)	1250 (900,1600)	1200 (900,1600)	0.001	1250 (900,1600)	1300 (900,1700)	−0.195
Fluid management grade			0.251			0.041
Low fluid intake	54 (22.0)	388 (30.8)		54 (22.0)	180 (24.4)	
Moderate fluid intake	145 (58.9)	718 (57.1)		145 (58.9)	435 (58.9)	
High fluid intake	47 (19.1)	152 (12.1)		47 (19.1)	123 (16.7)	
Blood loss (mL)	271.10 ± 146.64	235.11 ± 136.99	0.239	271.10 ± 146.64	261.96 ± 142.23	0.064

To reduce baseline bias and enhance comparability between groups, several matching strategies were evaluated. A 1:3 nearest-neighbor approach with a caliper of 0.2 provided the most favorable balance between covariate comparability and statistical efficiency and was selected for the primary analysis (Supplementary Material 3). This method retained all malnourished patients and yielded a matched control cohort of 738 individuals. Covariate balance before and after matching is also presented in Table 1.

**Fig. 1:**
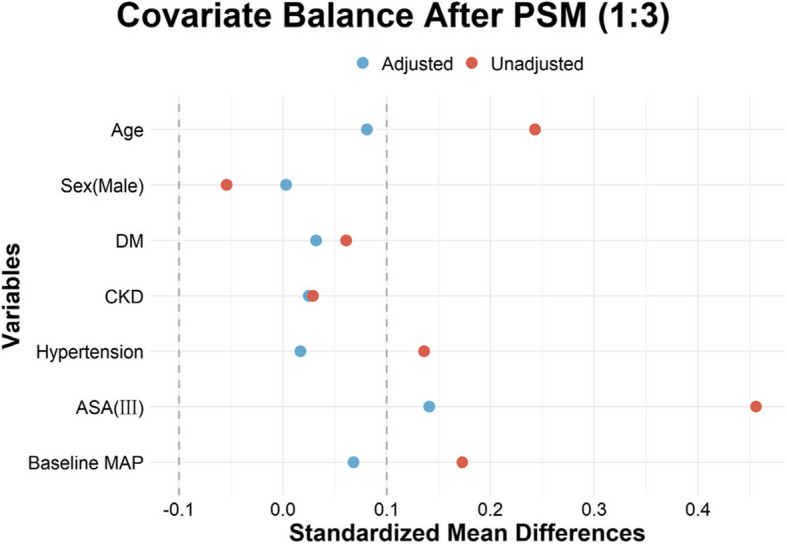
Covariate balance of variables included in propensity score matching before and after matching. Standardized mean differences are shown for each covariate before (red) and after (blue) matching. Matching substantially reduced imbalances across all variables, indicating adequate covariate balance. PSM = propensity score matching; SMD = standardized mean difference; DM = diabetes mellitus; CKD = chronic kidney disease; ASA = American Society of Anesthesiologists; MAP = mean arterial pressure

Covariates for the propensity score model were selected based on clinical judgment and supporting evidence from prior literature (Schnetz et al. 2022; Scott 2024), including age, sex, diabetes mellitus (DM), chronic kidney disease (CKD), hypertension, ASA physical status, and baseline mean arterial pressure (MAP) (Fig.1).

While only selected preoperative variables were included in the matching model, additional baseline and perioperative characteristics were assessed using standardized mean differences (SMDs) to provide a sample size–independent evaluation of group comparability (Table 1). Notably, BMI and serum albumin—excluded from the matching model due to their diagnostic role—showed residual moderate imbalance after matching. Most perioperative variables were well balanced, with small residual imbalances noted in total fluid volume and sufentanil dosage.

### Multidimensional assessment of the primary outcome: IOH

Table 2 presents the comparative analysis of IOH between malnourished and non-malnourished patients, across four predefined dimensions: incidence, cumulative duration, time proportion, and severity (lowest MAP).

**Table 2:** Effect of nutritional status on multiple dimensions of intraoperative hypotension

Variables	Malnutrition Yes (*n* = 246)	Malnutrition No (*n* = 738)	*P*-value	Effect size
Absolute IOH				
Incidence^a^	43 (17.5)	122 (16.5)	0.805	0.03 (−0.05–0.10)
Duration (mins)^b^	0 (0,0)	0 (0,0)	0.768	0.01 (−2.60–2.63)
Proportion^b^	0 (0,0)	0 (0,0)	0.885	0.01 (−2.60–2.63)
Relative IOH				
Incidence^a^	246 (100.0)	726 (98.4)	0.044	0.26 (0.15–0.36)
Duration (mins)^c^	89.15 ± 38.68	71.99 ± 39.8	< 0.001	0.43 (0.29–0.58)
Proportion^c^	0.48 ± 0.16	0.39 ± 0.17	< 0.001	0.54 (0.39–0.69)
Lowest MAP (mmHg)^c^	69.84 ± 6.24	70.81 ± 7.03	0.041	−0.14 (−0.29–0.00)

^a^Cohen’s *h*

^b^Cliff’s delta

^c^Cohen’s *d*

For absolute IOH, there was no meaningful difference between the two groups in any dimension. The incidence was nearly identical (17.5% vs. 16.5%, *P* = 0.805), and both the duration and proportion of time spent in absolute IOH were similar (Supplementary Material 4). The effect sizes across all comparisons were negligible, indicating a lack of clinically relevant difference.

In contrast, relative IOH demonstrated consistent differences in favor of greater hemodynamic compromise in the malnourished group. Specifically:

- The incidence was modestly higher (Cohen’s *h* = 0.26, *P* = 0.044);
- The duration of hypotension was longer (Cohen’s *d* = 0.43, *P* < 0.001);
- The proportion of anesthesia time spent in hypotension was greater (Cohen’s *d* = 0.54, *P* < 0.001);
- The lowest MAP was slightly lower (Cohen’s *d* = −0.14, *P* = 0.041).

Multiple dimensions of IOH—including incidence, cumulative duration, lowest MAP, and the proportion of anesthesia time spent in relative hypotension—were assessed in the matched cohort. These metrics yielded varying results: while some showed statistically significant group differences, their effect sizes were modest; others demonstrated no meaningful difference. This variability likely reflects inherent limitations of the retrospective design, such as residual confounding or inconsistencies in intraoperative management, as further discussed in the “Discussion” section.

Among these, the proportion of anesthesia time spent in relative IOH was designated a priori as the primary outcome for exploratory multivariable analyses. This continuous measure provides higher resolution and sensitivity in quantifying cumulative hemodynamic burden and offers superior clinical interpretability, making it the most appropriate metric for subsequent modeling.

### Model performance and predictive accuracy

Given that the primary outcome of the exploratory multivariable analysis—the proportion of IOH—is a continuous variable bounded between 0 and 1, a beta regression model was employed to appropriately account for its distributional characteristics.

The final model demonstrated a good overall fit, with an AIC of −1440.38 and a BIC of −1386.57. A pseudo *R*^2^ value of 0.3364 indicated that the model explained approximately 33.6% of the variability in the proportion of IOH, suggesting moderate-to-strong explanatory power.

Model calibration was acceptable, as evidenced by a low Brier score of 0.0107 (ideal value < 0.1), indicating minimal prediction error and strong predictive accuracy (see Supplementary Material 5).

Based on its explanatory strength, goodness of fit, and predictive reliability, this model was selected for the subsequent multivariable analysis presented in the next section.

### Multivariable analysis of intraoperative hypotension: focus on the proportion of time in relative IOH

In the multivariable beta regression model (Table 3, Supplementary Material 6), malnutrition remained a significant and independent predictor of a higher proportion of time spent in relative intraoperative hypotension (*P* < 0.001), even after adjusting for age, sex, albumin, CRP, and other relevant covariates.

**Table 3:** Effect of malnutrition on the primary outcome: beta regression analysis of the proportion of time in relative intraoperative hypotension

Variables	Estimate	Standard error	*Z*-score	*P*-value	95% CI
Malnutrition	0.303	0.063	4.80	< 0.001	[0.179, 0.427]
Age	0.021	0.002	12.34	< 0.001	[0.117, 0.204]
Sex (male)	−0.123	0.038	−3.24	0.001	[−0.197, −0.049]
Albumin	−0.014	0.005	−2.73	0.006	[−0.023, −0.004]
CRP	0.007	0.003	2.59	0.010	[0.002, 0.013]
Laparoscopic surgery	1.147	0.054	21.33	< 0.001	[1.042, 1.252]
Surgery duration	−0.002	0.001	−2.52	0.012	[−0.004, −0.001]
Baseline MAP	0.004	0.002	2.59	0.010	[0.001, 0.007]
Blood loss	0.002	0.001	12.09	< 0.001	[0.002, 0.003]

Additional factors associated with an increased proportion of relative IOH included older age, elevated CRP levels, laparoscopic surgery, higher preoperative MAP, and greater intraoperative blood loss. In contrast, male sex, higher serum albumin concentrations, and longer operative duration were associated with a lower IOH proportion.

This association remained robust across a series of robustness checks, including stratified and IPTW analyses (Supplementary Material 7) as well as an additional analysis using ward-based baseline MAP (Supplementary Material 8), all of which yielded consistent findings.

## Discussion

This retrospective cohort study demonstrated that preoperative malnutrition is associated with a greater intraoperative hemodynamic burden, as reflected across multiple dimensions of relative hypotension. While the degree of difference varied among metrics—including incidence, cumulative duration, and lowest MAP—the proportion of anesthesia time spent in relative hypotension showed the most pronounced and clinically interpretable between-group difference. Accordingly, this parameter was pre-specified as the primary outcome for multivariable modeling.

In the subsequent beta regression analysis, malnutrition remained a significant and independent predictor of increased relative IOH proportion after adjusting for age, sex, albumin, CRP, surgical approach, baseline MAP, and intraoperative factors. These findings reinforce the inference that malnutrition compromises hemodynamic resilience during surgery.

The observed association may be explained by several pathophysiological mechanisms. Malnutrition can impair autonomic cardiovascular regulation, reduce metabolic reserves, and compromise vascular tone. Hypoalbuminemia may decrease oncotic pressure and intravascular volume (Sganzerla et al. 2025; Sicova et al. 2024; van de Wouw and Joles 2022), while elevated CRP levels reflect chronic systemic inflammation, which may affect vascular reactivity and endothelial function (Wachtendorf et al. 2022; Wang et al. 2024; Widlansky 2003). These mechanisms are consistent with prior observations linking nutritional status to hemodynamic instability in cardiac surgery (Wiedermann 2022), orthostatic intolerance (Lin et al. 2021), pregnancy-associated circulatory changes (Lubrano et al. 2024), and critical illness (Maiwall et al. 2022). Collectively, these clinical observations support the plausibility of our findings, highlighting a consistent pattern of impaired hemodynamic adaptability in malnourished states across various physiological and surgical contexts.

Although our study demonstrated a significant association between malnutrition and relative hypotension, no significant differences were observed between groups in terms of absolute hypotension. This discrepancy may be attributed to differences in clinical management and the inherent sensitivity of the respective measures. Absolute hypotension, typically defined as MAP < 65 mmHg, often triggers immediate anesthetic interventions—such as fluid administration or vasopressor use—which can obscure between-group differences. In contrast, relative hypotension reflects reductions from each patient’s baseline MAP and captures the cumulative burden of even mild hemodynamic compromise. It may therefore offer greater sensitivity in identifying hemodynamic vulnerability, especially in high-risk surgical patients, where moderate MAP declines may still carry clinical significance (Zeller et al. 2024).

Notably, the incidence of relative hypotension exceeded 98% in both groups. Although the difference was statistically significant—likely due to the large sample size—the effect size was small, indicating limited clinical relevance. However, this finding does not imply the absence of hemodynamic burden or clinical relevance. Rather, it reflects the inherent limitations of binary outcome variables in interpretability. In this study, relative hypotension was defined as any intraoperative MAP falling below 80% of a patient’s baseline value. This threshold is highly sensitive, capturing even brief or mild MAP reductions, and thereby resulting in near-universal classification of patients as hypotensive. While this enhances detection sensitivity, it substantially reduces discriminatory power and statistical interpretability, especially when event rates approach saturation. In contrast, using a continuous variable—the proportion of anesthesia time spent in relative hypotension—offers greater resolution and more accurately reflects the cumulative burden and severity of MAP declines. Thus, although no significant difference was found in binary incidence, its high prevalence still underscores the relevance of relative hypotension in this surgical population and justifies our subsequent analytical strategy.

To address potential concerns that the high incidence may have resulted from an artificially elevated baseline, we emphasize that baseline MAP was defined as the average of non-invasive blood pressure measurements obtained after operating room admission but prior to anesthetic induction. Although this may be higher than ward or resting MAP values, it more accurately reflects the physiological state immediately preceding surgical stress, and is therefore an appropriate comparator for intraoperative hemodynamic assessments. Moreover, a sensitivity analysis using ward-based MAP in a thoracic surgery subset yielded consistent results, further supporting the robustness of our findings.

Beyond the sensitivity analysis, a further methodological strength of this study is the use of PSM. Because malnourished patients often present with multiple comorbidities and higher baseline risk, direct comparisons could be biased by these differences. By balancing demographic and perioperative characteristics, PSM enabled a more accurate assessment of the independent association between malnutrition and intraoperative hypotension. Nevertheless, as with all observational studies, PSM cannot fully address unmeasured confounding, which should be taken into account when interpreting the findings.

Taken together, these findings underscore the importance of incorporating nutritional assessment into perioperative risk stratification, particularly among high-risk surgical patients. Clinicians should be aware of the increased susceptibility to hemodynamic instability in malnourished individuals and consider nutritional risk as part of comprehensive perioperative management. At the same time, this single-center retrospective observational study has inherent limitations that constrain causal inference. Intraoperative blood pressure data were extracted from an electronic anesthesia recording system with 5-min intervals, limiting temporal resolution and potentially missing transient but clinically relevant hypotensive episodes. Given this constraint, we did not use time-weighted average MAP, which requires high-frequency or continuous monitoring, but instead employed multiple complementary indicators—including lowest MAP, cumulative duration, and proportion of anesthesia time below threshold—to characterize intraoperative hypotension. These surrogate metrics were chosen for their data availability and clinical interpretability, allowing a multidimensional assessment of hemodynamic burden. Moreover, because anesthetic management strategies such as vasopressor use and anesthesia depth adjustments were individualized, not protocolized, and not routinely recorded at our center, potential intraoperative confounding—particularly affecting absolute hypotension—could not be fully accounted for. Taken together, these limitations warrant cautious interpretation of our findings. Future prospective studies with standardized intervention protocols, continuous data acquisition, and detailed intraoperative medication records are needed to confirm and extend our observations.

## Conclusions

In high-risk surgical patients, preoperative malnutrition was independently associated with an increased burden of intraoperative hemodynamic instability when assessed by relative blood pressure thresholds. This relationship was consistently observed across multiple analytical dimensions, supporting the integration of nutritional status into perioperative risk assessment frameworks. The findings also underscore the need for prospective investigations to guide more targeted perioperative management strategies. Although based on retrospective data, the present study provides a strong foundation for future validation of underlying mechanisms and the development of effective interventional approaches.

## Supplementary Information

Supplementary Material 1: Diagnostic Criteria for Severe Autonomic Dysfunction. Supplementary Material 2: Flowchart. Supplementary Material 3: Evaluation of Propensity Score Matching Strategies and Justification for the Selected 1:3 Weighted Matching Scheme. Supplementary Material 4: Effect of Nutritional Status on Severity of Hypotension in Patients with Absolute IOH. Supplementary Material 5: Calibration Curve and Predictive Accuracy of the Main Model. Supplementary Material 6: Regression Coefficient Plot for Primary Analysis. Supplementary Material 7: Robustness Checks of the Beta Regression Model: Stratified and Sensitivity Analyses. Supplementary Material 8: Robustness Check with Ward-Based Baseline MAP.

## Data Availability

The datasets used and analyzed during the current study are available from the corresponding author upon reasonable request.

## Abbreviations

ASA: American Society of Anesthesiologists
BMI: Body mass index
CKD: Chronic kidney disease
CRP: C-reactive protein
DM: Diabetes mellitus
eGFR: Estimated glomerular filtration rate
GLIM: Global Leadership Initiative on Malnutrition
IOH: Intraoperative hypotension
IABP: Invasive arterial blood pressure
MAP: Mean arterial pressure
NYHA: New York Heart Association
PSM: Propensity score matching
SMD: Standardized mean difference
