# Supplementary material for: Preoperative malnutrition is a risk factor for intraoperative hypotension in high-risk surgical patients: a propensity score–matched cohort study
Source: Perioper Med (Lond). 2025 Nov 19;14:130. doi: 10.1186/s13741-025-00620-x (PMC12628959; doi:10.1186/s13741-025-00620-x)
Supplement: Supplementary file 1 — Supplementary Material 1: Diagnostic Criteria for Severe Autonomic Dysfunction. Supplementary Material 2: Flowchart. Supplementary Material 3: Evaluation of Propensity Score Matching Strategies and Justification for the Selected 1:3 Weighted Matching Scheme. Supplementary Material 4: Effect of Nutritional Status on Severity of Hypotension in Patients with Absolute IOH. Supplementary Material 5: Calibration Curve and Predictive Accuracy of the Main Model. Supplementary Material 6: Regression Coefficient Plot for Primary Analysis. Supplementary Material 7: Robustness Checks of the Beta Regression Model: Stratified and Sensitivity Analyses. Supplementary Material 8: Robustness Check with Ward-Based Baseline MAP. [file 13741_2025_620_MOESM1_ESM.docx]

**Supplementary Material 1**. Diagnostic Criteria for Severe Autonomic Dysfunction

Severe autonomic dysfunction is diagnosed if any of the following criteria are met:

(1) ICD-10 Diagnosis Code Recorded in the Electronic Medical Record (Fulfilling Any of the Following Conditions):

-E11.43: Type 2 diabetes mellitus with autonomic neuropathy

- I95.1: Orthostatic hypotension

- G20: Parkinson’s disease with autonomic dysfunction

- G90.3: Multiple system atrophy with autonomic dysfunction

- G99.0: Diabetic autonomic neuropathy

- N31.9: Neurogenic bladder

- K31.84: Diabetic gastroparesis

(2) No ICD-10 Diagnosis Code but Fulfilling the Following Criteria:

-A diabetes mellitus duration of ≥10 years and at least one documented laboratory result indicating an HbA1c level >8.0%.

**Supplementary Material 2**. Flowchart


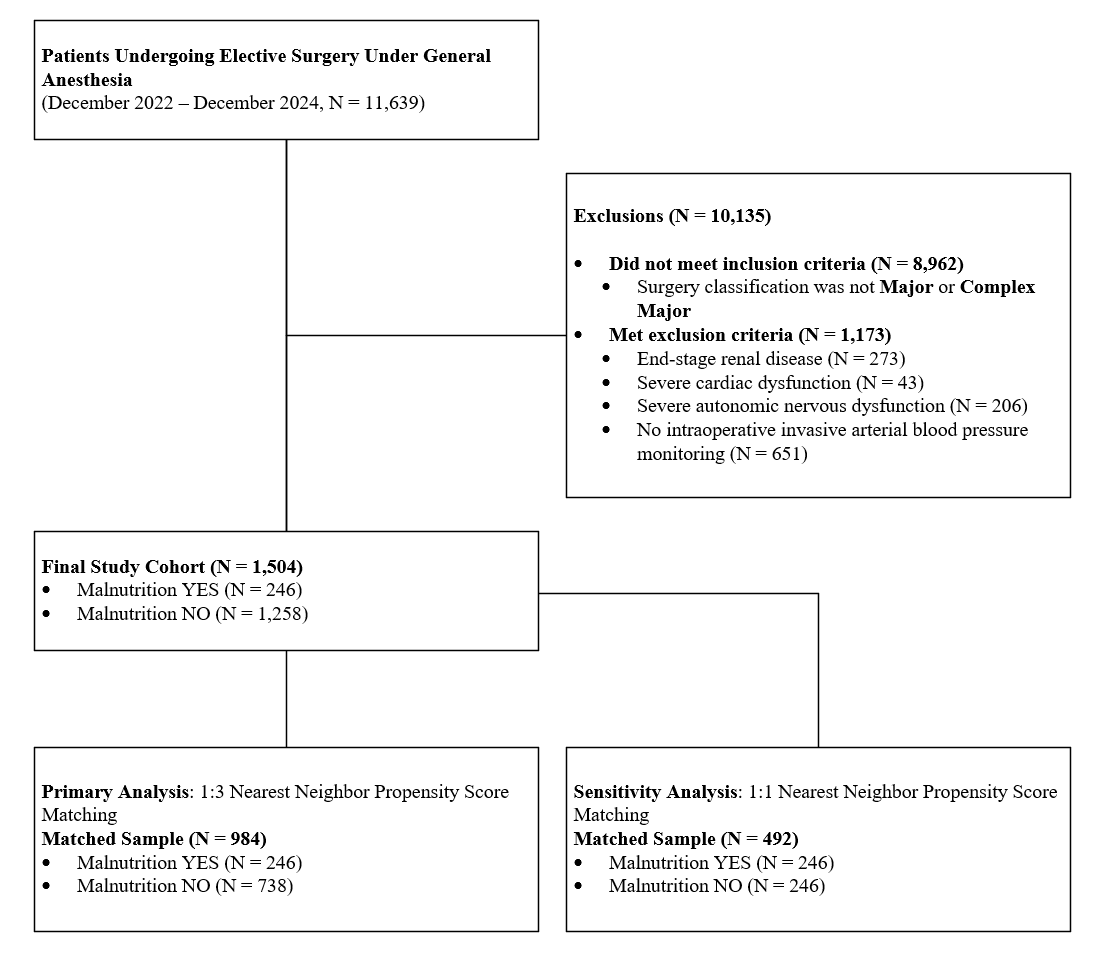


**Supplementary Material 3**. Evaluation of Propensity Score Matching Strategies and Justification for the Selected 1:3 Weighted Matching Scheme

Comparison of Propensity Score Matching Strategies and Covariate Balance Metrics

| Matching Strategy | Caliper Width | Matched Sample Size  (Malnutrition / Control) | Mean SMD | Max SMD |
| --- | --- | --- | --- | --- |
| 1 : 1 NN | 0.2 | 246 / 246 | 0.045 | 0.115 |
| 1 : 2 NN | 0.2 | 246 / 492 | 0.049 | 0.125 |
| 1 : 3 NN | 0.2 | 246 / 738 | 0.052 | 0.141 |
| 1 : 4 NN | 0.2 | 213 / 639 | 0.058 | 0.156 |
| 1 : 3 NN | 0.1 | 197 /591 | 0.038 | 0.098 |

Abbreviations: NN = nearest-neighbor; SMD = standardized mean difference; IPTW = inverse probability of treatment weighting; ESS = effective sample size.

Among all evaluated matching strategies, 1:3 nearest-neighbor matching with a caliper of 0.2 achieved the optimal trade-off between covariate balance (mean SMD = 0.052) and sample retention, preserving all malnourished patients (n = 246), and was therefore selected as the primary analytical approach. Although the stricter 1:3 matching with a caliper of 0.1 provided slightly better covariate balance, it resulted in the exclusion of approximately 20% of malnourished patients, thus compromising representativeness and statistical power.

While the control pool was sufficiently large to theoretically support up to 1:5 matching, we observed a decline in the number of matched malnourished patients beginning at the 1:4 strategy (n = 213), indicating that further increases in the matching ratio were infeasible under caliper constraints. As a result, 1:5 matching was not attempted.

Additionally, we applied inverse probability of treatment weighting (IPTW) using stabilized weights on the full sample. Although IPTW achieved favorable covariate balance (mean SMD = 0.050, maximum SMD = 0.138), it yielded a lower effective sample size (ESS = 817) and may be more susceptible to extreme weights and instability, particularly in subgroups with limited covariate overlap. Therefore, IPTW was not selected as the primary method but was employed as a sensitivity analysis to confirm the robustness of the main findings (see Supplementary Material 6).

**Supplementary Material 4**. Effect of Nutritional Status on Severity of Hypotension in Patients with Absolute IOH

| Variables | Malnutrition  YES（n=43） | Malnutrition  NO（n=122） | P-value | Effect Size |
| --- | --- | --- | --- | --- |
| Duration^c^ | 12.79(3.68) | 13.16(4.27) | 0.619 | -0.09(-0.44-0.26) |
| Proportion^b^ | 0.07(0.05-0.08) | 0.07(0.06-0.09) | 0.629 | -0.07(-0.08-0.06) |
| Lowest MAP^c^ | 62.65(2.48) | 62.72(2.68) | 0.881 | -0.03(-0.37-0.32) |

Abbreviations: b: Cliff's delta；c：Cohen’s d.

**Supplementary Material 5**. Calibration Curve and Predictive Accuracy of the Main Model


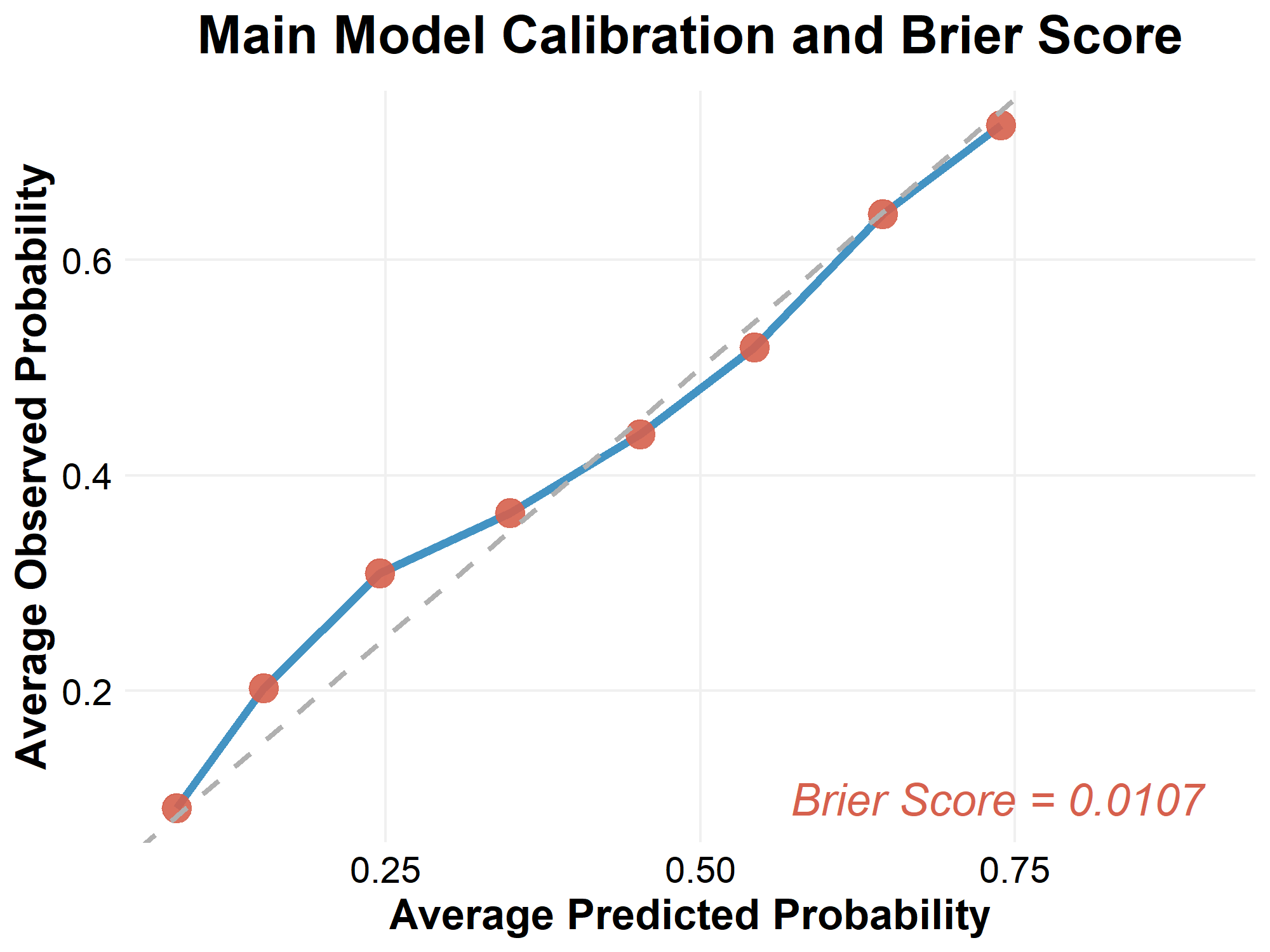


Figure S1. Calibration plot of the main prediction model based on the PSM-Matched dataset.

The main model refers to the beta regression model presented in the main text.

The x-axis represents the average predicted probability, and the y-axis shows the average observed probability across deciles.

The solid blue line indicates the actual calibration, while the dashed diagonal line represents perfect prediction.

The Brier score is 0.0107, indicating excellent calibration and low prediction error (ideal value < 0.1).

**Supplementary Material 6**. Regression Coefficient Plot for Primary Analysis


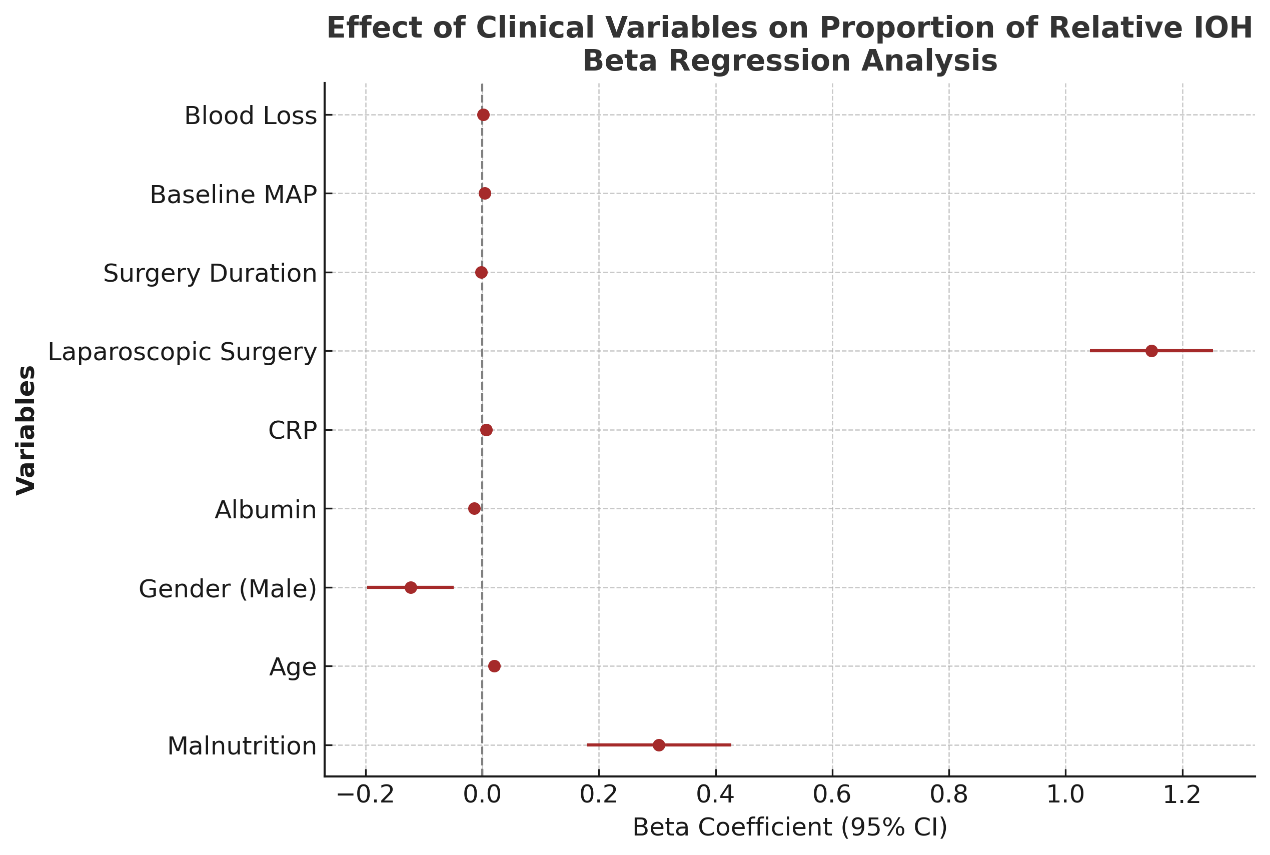


Figure S2. Regression coefficient plot from the primary analysis using beta regression.

This plot illustrates the estimated effects of selected clinical variables on the proportion of relative IOH.

Each dot represents the beta coefficient with 95% confidence interval (CI).

Variables with confidence intervals not crossing zero are considered statistically significant.

The model was constructed using the propensity score–matched dataset as described in the main analysis.

**Supplementary Material 7.** Robustness Checks of the Beta Regression Model: Stratified and Sensitivity Analyses

In the primary analysis, malnutrition remained significantly associated with the proportion of intraoperative hypotension (IOH) (β = 0.303, P < 0.001). This association was consistent across stratified analyses by surgical type and hypertension history. Notably, the effect of malnutrition was most pronounced among patients undergoing thoracic surgeries, suggesting that this subgroup may be particularly vulnerable to IOH due to compromised nutritional status.

Similarly, in the IPTW-weighted dataset, sensitivity analyses confirmed that malnutrition remained a significant predictor of IOH. Although the IPTW model yielded slightly poorer fit indices (AIC = −840.564 vs. −1440.377; BIC = −794.381 vs. −1386.569), its explanatory power (pseudo R² = 0.3982 vs. 0.3382) and predictive performance (Brier Score = 0.0082 vs. 0.0112) were comparable to those of the primary model. These findings support the robustness of the observed association across different analytical approaches and matching strategies.

Effect of Malnutrition on the Proportion of Relative Intraoperative Hypotension: Subgroup and Sensitivity Analyses of the Primary Outcome

| Sample / Subgroup | Estimate | Standard Error | Z-score | P-value | 95% CI |
| --- | --- | --- | --- | --- | --- |
| Main (1:3) | 0.303 | 0.063 | 4.80 | <.001 | [0.179, 0.427] |
| Abdominal (1:3) | 0.339 | 0.064 | 5.32 | <.001 | [0.214, 0.464] |
| Orthopedic (1:3) | 0.367 | 0.058 | 6.32 | <.001 | [0.253, 0.480] |
| Thoracic (1:3) | 0.812 | 0.248 | 3.27 | 0.001 | [0.326, 1.299] |
| Urological (1:3) | 0.668 | 0.087 | 7.67 | <.001 | [0.491, 0.839] |
| Hypertension (-) (1:3) | 0.460 | 0.060 | 7.70 | <.001 | [0.344, 0.576] |
| Hypertension (+) (1:3) | 0.529 | 0.070 | 7.57 | <.001 | [0.392, 0.664] |
| Sensitivity (IPTW) | 0.430 | 0.070 | 6.08 | <.001 | [0.291, 0.568] |

Abbreviations: IPTW = inverse probability of treatment weighting; CI = confidence interval.


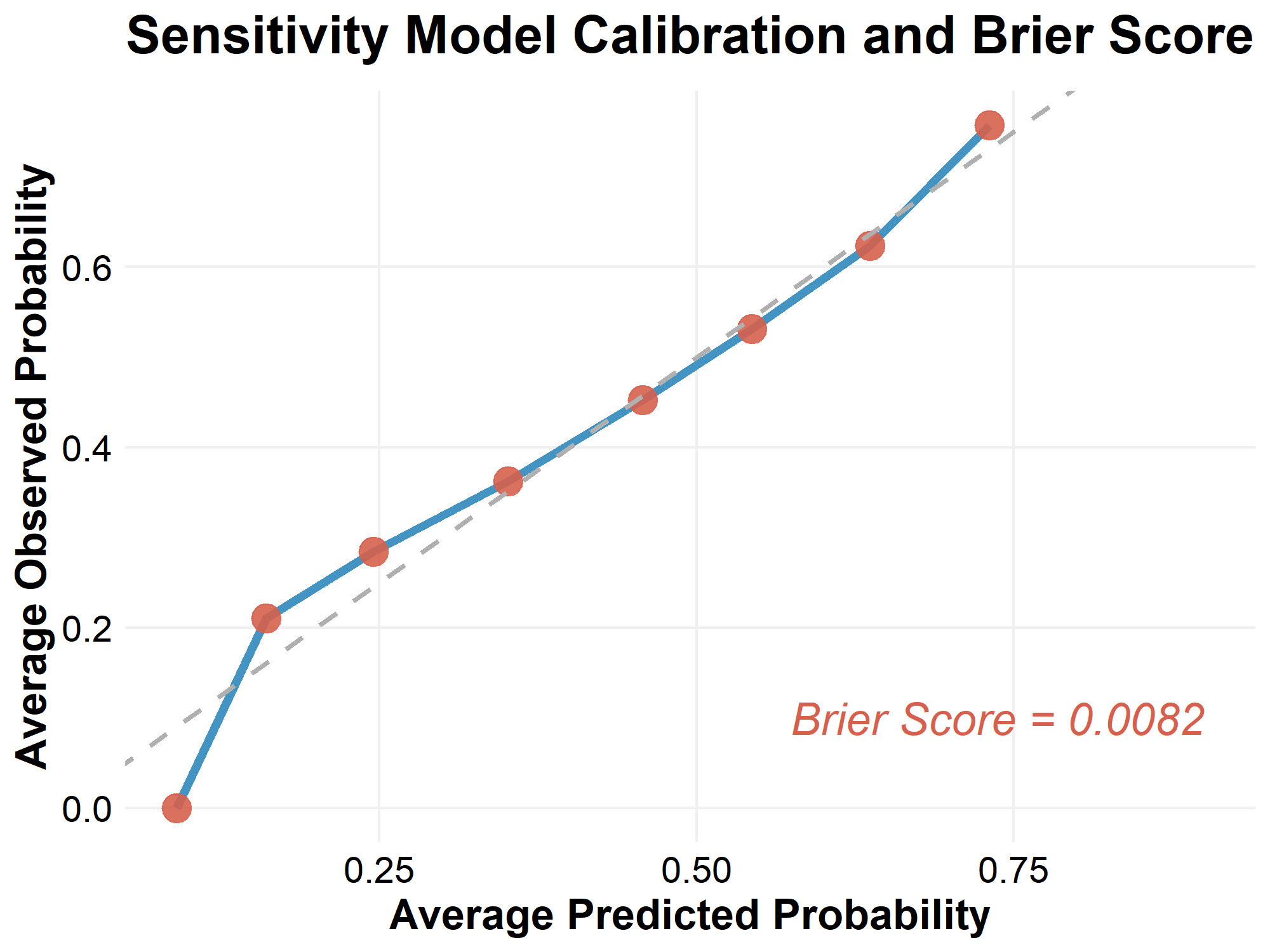


Figure S3. Calibration plot of the sensitivity model based on the IPTW-weighted dataset.

The sensitivity model was constructed using beta regression on the dataset weighted by inverse probability of treatment weighting (IPTW), as described in the sensitivity analysis.

The x-axis represents the average predicted probability, and the y-axis shows the average observed probability across deciles.

The solid blue line indicates the actual calibration, while the dashed diagonal line represents perfect prediction.

The Brier score is 0.0082, indicating excellent calibration and low prediction error (ideal value < 0.1).

**Supplementary Material 8.** Robustness Check with Ward-Based Baseline MAP

In this study, pre-anesthesia blood pressure was used as the baseline to capture patients’ physiological status immediately before induction. This approach is relatively standardized and reflects the hemodynamic state most relevant to anesthetic management. Nevertheless, blood pressure values obtained in the holding area may be artificially elevated by preoperative anxiety, which could lead to an overestimation of relative IOH and complicate interpretation. To address this concern and test the robustness of our findings, we conducted a supplementary analysis in a thoracic surgery subset with a sufficiently large sample size and consistently documented ward blood pressure measurements.

In this subset, ward blood pressure was measured by nursing staff using an automated oscillometric device and recorded in the medical charts, although details regarding patient position and exact timing were unavailable. MAP was calculated from all ward measurements obtained during the last two complete preoperative hospital days, and the median value was used as the baseline for each patient. Patients without ward recordings in this window were classified as missing. Using this alternative baseline, relative IOH events were redefined, and between-group differences were evaluated across three metrics: incidence, cumulative duration, and proportion of anesthesia time, with corresponding effect sizes calculated. Absolute IOH and intraoperative lowest MAP were not included, as their definitions do not depend on baseline choice. Finally, we reapplied the multivariable β-regression model from the main analysis to this subset to examine whether malnutrition remained an independent predictor of the proportion of time spent in relative IOH.

A total of 328 patients were included, of whom 22 had a hospital stay of less than two days but still had eligible ward blood pressure records and were therefore retained. The malnourished group comprised 82 patients and the non-malnourished group 246 patients. When ward-based values were used, baseline MAP decreased compared with holding-area measurements: from 107.84±11.18 to 94.59±7.14 mmHg in the malnourished group, and from 106.71±11.17 to 96.15±9.06 mmHg in the non-malnourished group. Between-group balance was preserved (SMD increased from 0.090 to 0.192, both <0.2, indicating acceptable balance).

Under this definition, the absolute incidence and duration of relative IOH were lower than those observed with the holding-area baseline. Nevertheless, malnourished patients continued to demonstrate a higher burden across all three dimensions: incidence 74.4% vs 57.3% (P=0.006; Cohen’s h=0.36), cumulative duration 50 (6.25–70) vs 35 (0–60) min (P=0.001; Cliff’s delta=0.23), and proportion of anesthesia time 0.30 (0.04–0.33) vs 0.21 (0–0.30) (P<0.001; Cliff’s delta=0.27).

In the multivariable β-regression analysis (dependent variable: proportion of time in relative IOH; covariates identical to the main analysis except for surgical type), malnutrition remained an independent predictor (β=0.452, 95% CI: 0.038–0.866, P=0.032).

Taken together, these results indicate that the association between malnutrition and intraoperative relative hypotension persisted when ward-based MAP was used to define the baseline, with consistent direction and comparable magnitude, thereby supporting the robustness of the main findings. Although the extent of change varied across the three IOH metrics, these differences did not materially affect the overall conclusion.
